# Evaluation of Antigens for Development of a Serological Test for Human African Trypanosomiasis

**DOI:** 10.1371/journal.pone.0168074

**Published:** 2016-12-09

**Authors:** Sylvain Biéler, Harald Waltenberger, Michael P. Barrett, Richard McCulloch, Jeremy C. Mottram, Mark Carrington, Wilhelm Schwaeble, James McKerrow, Margaret A. Phillips, Paul A. Michels, Philippe Büscher, Jean-Charles Sanchez, Richard Bishop, Derrick R. Robinson, James Bangs, Michael Ferguson, Barbara Nerima, Audrey Albertini, Gerd Michel, Magdalena Radwandska, Joseph Mathu Ndung’u

**Affiliations:** 1 Foundation for Innovative New Diagnostics (FIND), Geneva, Switzerland; 2 Microcoat Biotechnologie GmbH, Bernried, Germany; 3 Wellcome Trust Centre for Molecular Parasitology, University of Glasgow, Glasgow, United Kingdom; 4 University of Cambridge, Cambridge, United Kingdom; 5 University of Leicester, Leicester, United Kingdom; 6 University of California, San Francisco, California, United States of America; 7 University of Texas Southwestern Medical Center, Dallas, Texas, United States of America; 8 Christian de Duve Institute, Brussels, Belgium; 9 Institute of Tropical Medicine, Antwerp, Belgium; 10 Biomedical Proteomics Research Group, University of Geneva, Geneva, Switzerland; 11 International Livestock Research Institute, Nairobi, Kenya; 12 CNRS UMR-5234, University of Bordeaux, Bordeaux, France; 13 University of Wisconsin-Madison, Madison, Wisconsin, United States of America; 14 University of Dundee, Dundee, United Kingdom; 15 University of Bern, Bern, Switzerland / Makerere University, Kampala, Uganda; Yeshiva University Albert Einstein College of Medicine, UNITED STATES

## Abstract

**Background:**

Control and elimination of human African trypanosomiasis (HAT) can be accelerated through the use of diagnostic tests that are more accurate and easier to deploy. The goal of this work was to evaluate the immuno-reactivity of antigens and identify candidates to be considered for development of a simple serological test for the detection of *Trypanosoma brucei gambiense* or *T*. *b*. *rhodesiense* infections, ideally both.

**Methodology/Principal Findings:**

The reactivity of 35 antigens was independently evaluated by slot blot and ELISA against sera from both *T*. *b*. *gambiense* and *T*. *b*. *rhodesiense* infected patients and controls. The antigens that were most reactive by both tests to *T*. *b*. *gambiense* sera were the membrane proteins VSG LiTat 1.3, VSG LiTat 1.5 and ISG64. Reactivity to *T*. *b*. *rhodesiense* sera was highest with VSG LiTat 1.3, VSG LiTat 1.5 and SRA, although much lower than with *T*. *b*. *gambiense* samples. The reactivity of all possible combinations of antigens was also calculated. When the slot blot results of 2 antigens were paired, a VSG LiTat 1.3- ISG75 combination performed best on *T*. *b*. *gambiense* sera, while a VSG LiTat 1.3-VSG LiTat 1.5 combination was the most reactive using ELISA. A combination of SRA and either VSG LiTat 1.3 or VSG LiTat 1.5 had the highest reactivity on *T*. *b*. *rhodesiense* sera according to slot blot, while in ELISA, pairing SRA with either GM6 or VSG LiTat 1.3 yielded the best results.

**Conclusions:**

This study identified antigens that were highly reactive to *T*. *b*. *gambiense* sera, which could be considered for developing a serological test for *gambiense* HAT, either individually or in combination. Antigens with potential for inclusion in a test for *T*. *b*. *rhodesiense* HAT were also identified, but because their reactivity was comparatively lower, a search for additional antigens would be required before developing a test for this form of the disease.

## Introduction

Human African trypanosomiasis (HAT) is a neglected tropical disease targeted by the World Health Organization (WHO) for elimination by 2020 [1]. Since the late 1990’s its global incidence has been declining steadily, but it continues to plague impoverished populations in a number of sub-Saharan African countries. Approximately 70 million people are estimated to be at risk of contracting the disease, which is generally fatal in the absence of proper diagnosis and treatment [2]. Two parasite sub-species are responsible for distinct forms of HAT. While *Trypanosoma brucei gambiense* causes a chronic disease in central and western Africa, *T*. *b*. *rhodesiense* causes acute infections in eastern Africa.

Diagnosis of *gambiense* HAT is routinely performed following algorithms that include screening to identify suspects, confirmation of disease, and staging to guide the choice of treatment [3]. Screening is an important process, which ensures that relatively complex and labour-intensive parasitological tests for confirmation are only performed on individuals who exhibit an immune response to the pathogen. The card agglutination test for trypanosomiasis (CATT/*T*.*b*. *gambiense*) has been the most widely used screening test for *gambiense* HAT over more than three decades. It detects host antibodies using as antigen a freeze dried suspension of purified, fixed and stained bloodstream form trypanosomes expressing LiTat 1.3 variant surface glycoprotein (VSG), which is a predominant variant antigen of *T*. *b*. *gambiense* [4]. However, CATT has a number of operational limitations that hinder its large-scale implementation, especially in basic health facilities in remote areas, including the need for specialized equipment, electricity and refrigeration. The sensitivity and specificity of CATT have also been reported to be sub-optimal in a number of settings [5]. To try and address some of these shortcomings, other screening tests have been developed. These include the LATEX/*T*. *b*. *gambiense* test, which is a card agglutination test similar to CATT but whose antigenic basis is a mixture of three purified variant surface glycoproteins (LiTat 1.3, 1.5 and 1.6) adsorbed on latex beads [6]. While further evaluations will be needed, currently available results indicate that the LATEX/*T*. *b*. *gambiense* test would have a higher specificity but a lower or similar sensitivity to the CATT test [5]. Immunofluorescence assays and enzyme-linked immunosorbent assay (ELISA) methods have also been used with success, but the sophisticated equipment that they require has resulted in their use being limited to reference laboratories [5]. Attempts have also been made to develop an antigen detection test for *gambiense* HAT, which would allow a distinction between past and current infections. These include the card indirect agglutination test for trypanosomiasis (TrypTect CIATT), which was found to be highly sensitive but whose specificity remains uncertain [5,7,8]. The situation is more problematic in countries that are endemic for *rhodesiense* HAT, as no screening test is available for this form of the disease.

Thus, developing and introducing new screening tools that would be both simple to use and highly accurate could play a central role in enhancing control and facilitating elimination of HAT. The goal of this study was to evaluate the diagnostic potential of antigens supplied by various research organisations and universities, and identify the most promising ones that could be used for development of a simple serological test for HAT. The test would ideally be a lateral-flow rapid diagnostic test (RDT) for screening patients suspected of HAT and populations living in endemic areas, which could be deployed within various levels of the healthcare system in HAT endemic countries, including rural, resource-limited settings where the disease is usually found.

## Materials and Methods

### Antigens

Candidate antigens were identified based on a literature search and through personal contacts. A request for access to antigens was sent to the corresponding academic organizations, and after signature of Material Transfer Agreements with FIND (Foundation for Innovative New Diagnostics), the antigens were shipped by the different suppliers to FIND where they were stored at -20°C. After all antigens were received, they were delivered to the University of Geneva Hospitals, Switzerland, where they were thawed and transferred into new tubes that were labelled with identification codes. Subsequently, the antigens, which were now anonymous as to function and origin, were shipped to Microcoat Biotechnologie GmbH where the screening was performed. Microcoat did not have access to any information that could allow identification of the antigens. Table 1 shows the 35 antigens that were screened in this study, including their source, supplier and method of production. Two of the antigens were purified native VSGs obtained from rodent infections, while the rest were purified recombinant proteins expressed in *Escherichia coli*.

**Table 1:** Antigens that were screened in this study.

Antigen name (abbreviation)	Source	Supplier	Reference
Phosphofructokinase (PFK)	*Trypanosoma brucei brucei*	Christian de Duve Institute	[9]
Fructose-1, 6-bisphosphate aldolase (ALD)	*Trypanosoma brucei*	Christian de Duve Institute	[10]
Triosephosphate isomerase (TIM)	*Trypanosoma brucei*	Christian de Duve Institute	[11]
Glyceraldehyde-3-phosphate dehydrogenase (GAPDH)	*Trypanosoma brucei*	Christian de Duve Institute	[12]
Phosphoglycerate kinase (PGK)	*Trypanosoma brucei*	Christian de Duve Institute	[13]
Phosphoglycerate mutase (PGAM)	*Trypanosoma brucei*	Christian de Duve Institute	[14]
Enolase (ENO)	*Trypanosoma brucei*	Christian de Duve Institute	[15]
Glucose-6-phosphate dehydrogenase (G6PDH)	*Trypanosoma brucei*	Christian de Duve Institute	[16]
Fructose-1,6-bisphosphatase (FBPase)	*Trypanosoma brucei*	Christian de Duve Institute	[17]
GM6 (GM6)	*Trypanosoma brucei*	International Livestock Research Institute	[18]
Glutathione S-transferase P1 (GSTP1)	Human	University of Geneva	[19]
Invariant Surface Glycoprotein 64–1 (ISG64)	*Trypanosoma brucei*	University of Cambridge	[20]
Invariant Surface Glycoprotein 65–1 (ISG65)	*Trypanosoma brucei*	University of Cambridge	[20]
Invariant Surface Glycoprotein 75–1 (ISG75)	*Trypanosoma brucei*	University of Cambridge	[20]
Serum resistance associated protein (SRA)	*Trypanosoma brucei rhodesiense*	University of Cambridge	[21,22]
Variant Surface Glycoprotein LiTat 1.3 (VSG LiTat 1.3)†	*Trypanosoma brucei gambiense*	Institute of Tropical Medicine	[23]
Variant Surface Glycoprotein LiTat 1.5 (VSG LiTat 1.5)†	*Trypanosoma brucei gambiense*	Institute of Tropical Medicine	[23]
6-phosphogluconate dehydrogenase (6PGDH)	*Trypanosoma brucei*	University of Glasgow	[24]
Primase small subunit (PriS)	*Trypanosoma brucei*	University of Glasgow	[25]
Rad51-4	*Trypanosoma brucei*	University of Glasgow	[26]
Calreticulin delta (1–25) (CRT-D)	*Trypanosoma cruzi*	University of Leicester	[27]
Calreticulin R-domain (CRT-R)	*Trypanosoma cruzi*	University of Leicester	[27]
Rhodesain/TbrCatL	*Trypanosoma brucei rhodesiense*	University of California	[28]
Ornithine decarboxylase (ODC)	*Trypanosoma brucei*	University of Texas Southwestern Medical Center	[29]
S-adenosylmethionine decarboxylase (AdoMetDC)	*Trypanosoma brucei*	University of Texas Southwestern Medical Center	[29,30]
Dihydroorotate dehydrogenase (DHODH)	*Trypanosoma brucei*	University of Texas Southwestern Medical Center	[31]
γ-glutamylcystein synthetase (GCS)	*Trypanosoma brucei*	University of Texas Southwestern Medical Center	[29]
Acyl-coA binding protein (ACBP)	*Trypanosoma brucei*	University of Dundee	[32]
70 kilodalton heat shock protein (HSP70)	*Trypanosoma brucei*	University of Wisconsin-Madison	[33]
Trypanopain/TbbCatL	*Trypanosoma brucei gambiense*	University of Wisconsin-Madison	[34]
Paraflagellar rod protein 2 (PFR2)	*Trypanosoma brucei*	University of Bordeaux	[35]
Inhibitor of cysteine peptidase (ICP)	*Trypanosoma brucei*	University of Glasgow	[36]
MARP1*	*Trypanosoma brucei*	University of Bern	[37]
Tb09.211.1800 (L14-6)*	*Trypanosoma brucei rhodesiense*	University of Bern	Nerima B (unpublished)
Tb10.70.6570 (16–6)*	*Trypanosoma brucei rhodesiense*	University of Bern	Nerima B (unpublished)

Except for the native antigens that were purified from rodent infections (†), all others were recombinant antigens produced using an E. coli expression system. Antigens marked with an asterisk (*) were only included in the third round of screening.

### Serum samples

Three rounds of screening were carried out, and for each round a different set of positive serum samples from *T*. *b*. *gambiense* and *T*. *b*. *rhodesiense* HAT cases, and negative controls were used. *T*. *b*. *gambiense* HAT cases were defined as patients who were positive by CATT and positive by any of the microscopy methods in routine use. *T*. *b*. *rhodesiense* HAT cases were defined as positive by any of the microscopy methods in routine use. Controls were negative by CATT (for samples collected in *T*. *b*. *gambiense* endemic regions) and by microscopy.

For the first round of screening, serum samples from 40 *T*. *b*. *gambiense* HAT cases (collected between 2002 and 2004) and 60 controls (including 50 samples collected in 2004 from HAT endemic regions and 10 from European donors) were supplied by the Institute of Tropical Medicine (ITM, Antwerp, Belgium). Serum samples from 10 *T*. *b*. *rhodesiense* HAT cases collected between 1990 and 2003 in Uganda were supplied by the National Livestock Resources Research Institute (NALIRRI, Tororo, Uganda); due to logistical challenges, these samples were thawed during transport and arrived at Microcoat at room temperature. For the second round, the same controls were used as in the first round, while NALIRRI supplied new sera from 35 *T*. *b*. *gambiense* HAT (collected in Uganda between 1998 and 2006) and 20 *T*. *b*. *rhodesiense* HAT cases (collected in Uganda between 2003 and 2008). Western blot and probing with an anti-human IgG conjugate revealed that nine of the *T*. *b*. *gambiense* HAT samples that were obtained from NALIRRI for the second round were significantly degraded, but these were kept in the study. The third round of screening was carried out using serum samples that were collected in 2008 and 2009 from 150 *T*. *b*. *gambiense* HAT cases, 33 *T*. *b*. *rhodesiense* HAT cases and 143 controls from a *T*. *b*. *gambiense* endemic region, which were obtained from the HAT Specimen Bank of the WHO.

Serum samples from the HAT Specimen Bank of the WHO were collected after approval by the WHO Research Ethics Review Committee and written informed consent from all participants. The samples from HAT endemic countries supplied by ITM were collected in 2004 during routine screening of patients by the national HAT control programs of the Democratic Republic of the Congo (DRC) and of Benin, for which ethical approval or informed consent was not required. The samples from European donors supplied by ITM were obtained in 2002 from the Red Cross for development of an RDT, and no information about ethical clearance could be found. The samples from NALIRRI were collected in Uganda between 1990 and 2008, and information about ethical approval for their collection could not be found.

### Slot blot

Slot blotting was used to test the reactivity of antigens as this method uses a matrix (nitrocellulose) that is close to the membrane of an RDT and is therefore expected to provide information that is directly related to the performance of an RDT. One μg of each antigen was transferred onto a 0.2 μm nitrocellulose membrane (Protran BA 83, Schleicher & Schüll) using a filtration manifold system (Whatman). The membrane was washed twice with Tris-buffered saline (TBS) pH 7.6 and incubated in blocking buffer (TBS, 1% PVP, 0.1% Tween 20, 5% bovine serum albumin) for 1.5 hours at room temperature. The serum sample was diluted 1:250 in phosphate buffered saline (PBS) supplemented with 5 mg/ml *E*. *coli* lysate and left to stand for 30 minutes (15 minutes in the first round of screening) at room temperature. The membrane was then incubated with the diluted serum for 1 hour at room temperature, and washed 3 times for 5 minutes in TBS-T (TBS supplemented with 0.1% Tween 20). This was followed by incubation for 30 minutes at room temperature with a goat anti-human IgG (Jackson Immuno Research Nr. 109-036-098) or IgM (Jackson Immuno Research Nr. 109-035-129) horseradish peroxidase (HRP) conjugate diluted at 0.01 μg/ml (0.02 μg/ml in the first round of screening) in blocking buffer. It was then washed 3 times for 5 minutes in TBS-T and 3 times for 5 minutes in water. Finally, the membrane was incubated for 3 minutes at 21°C with precipitating tetramethylbenzidine (TMB) (Sigma) and washed with water. The resulting reaction bands were analysed in duplicate using a flatbed scanner and the Tina 2.09 software, and the ratio of the densitometric value (optical density/mm^2^) of each reactive band to the background calculated. The three rounds of testing were performed in 2007, 2008 and 2009, respectively.

### ELISA

In addition to slot blot, ELISA testing was included as this method is more suitable in providing accurate quantitative data. Polystyrene microplates (Nunc MaxiSorp) were coated by adding 120 μl of antigen at a concentration of 10 μg/ml into each well and incubating for 16 hours at 20°C without shaking. They were then washed with PBS and incubated with blocking buffer (2.5% bovine serum albumin, 2.5% casein in PBS) for 1 hour at room temperature with shaking. After pre-incubating with *E*. *coli* lysate as described above, 100 μl of a serum sample diluted 1:250 in PBS was added and incubated for 1 hour at room temperature with shaking. The plates were washed three times with 300 μl/well of TBS-T, then 100 μl of anti-human IgG HRP conjugate diluted 1:160,000 in blocking buffer was added and incubated for 30 minutes at room temperature with shaking. This was followed by three rounds of washing with TBS-T at 300 μl/well, then 100 μl of TMB was added and incubated for 10 minutes at room temperature with shaking. The reaction was stopped by adding 50 μl of 1M H_2_SO_4_ and incubating for 1 minute at room temperature with shaking. The optical density was measured at 450 and 690 nm. The antigens were evaluated based on the absolute value of the signal as long as the signal to noise ratio was above 5:1, and otherwise were recorded as having no reactivity.

### Data analysis

An individual cut-off for each antigen and for each method (slot blot or ELISA) was calculated as the mean value obtained using all the control sera plus two standard deviations. The reactivity of each antigen was expressed as the percentage of samples from HAT cases for which the value was above the cut-off. Antigens were analyzed in duplicate, and the mean value recorded. In addition, Receiver Operating Characteristic (ROC) analysis was performed using the MedCalc statistical software (MedCalc Software bvba) to compute the sensitivity and specificity of each antigen by varying the cut-off. Youden’s index was calculated to evaluate accuracy [38].

## Results

**Fig 1:**
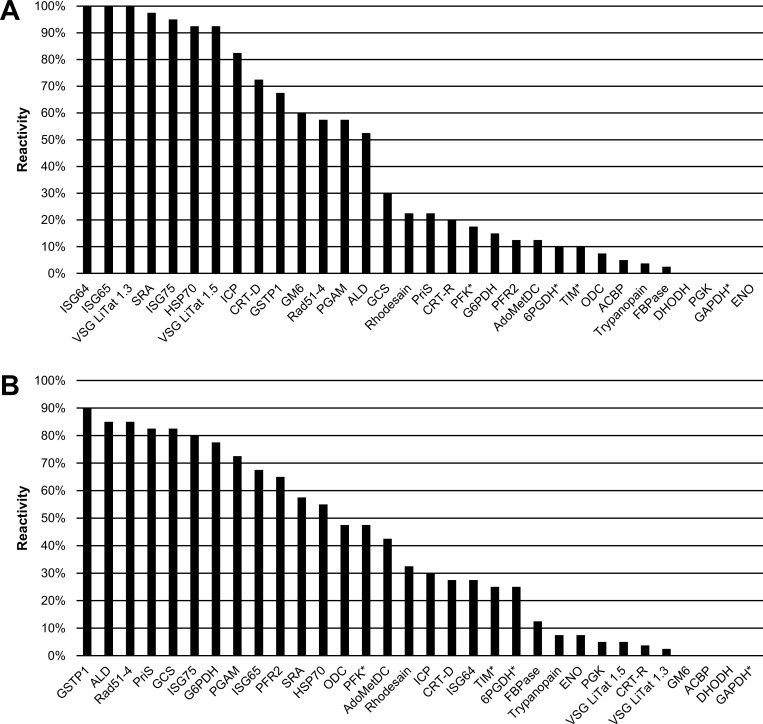
**Percentage IgG (A) and IgM (B) reactivity of 32 antigens on sera from *T*. *b*. *gambiense* HAT cases (N = 40) assessed by slot blot (first round of screening).** Antigens are shown in descending order of reactivity. Antigens marked with an asterisk (*) were only partially soluble.

The reactivity of 32 antigens against IgG and IgM in samples from HAT cases was evaluated by slot blotting in the first round of screening (Fig 1). There was a significant difference in the IgG and IgM reactivity of samples from *T*. *b*. *gambiense* cases. While the three most reactive antigens to IgG were ISG64 (100%), ISG65 (100%) and VSG LiTat 1.3 (100%), IgM reactivity was highest to GSTP1 (90%), ALD (85%) and Rad51-4 (85%). The three antigens that performed best with IgG (100%) only ranked 9^th^, 19^th^ and 28^th^ with IgM. When the 14 best antigens were compared, the percentage reactivity was higher with IgG than with IgM (9% higher on average).

**Fig 2:**
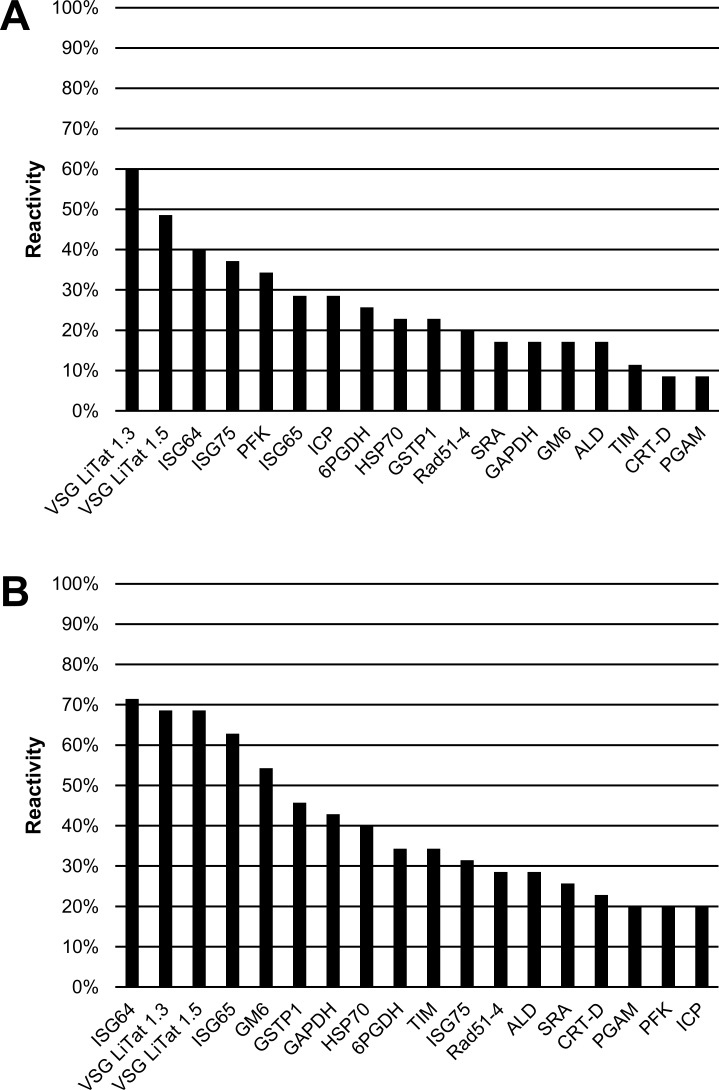
**Percentage IgG (A) and IgM (B) reactivity of 32 antigens on sera from *T*. *b*. *rhodesiense* HAT cases (N = 10) assessed by slot blot (first round of screening).** Antigens are shown in descending order of reactivity. Antigens marked with an asterisk (*) were only partially soluble.

The results of testing the antigens on *T*. *b*. *rhodesiense* HAT samples are shown in Fig 2. Two of the antigens that performed best in reactivity to either IgG or IgM (ISG64 and ISG65, and GSTP1 and ALD, respectively) were also the best on *T*. *b*. *gambiense* HAT samples. The performance of other less reactive antigens exhibited some differences when the results of the two sets of HAT sera were compared, although the overall ranking was quite similar. The reactivity was on average higher with *T*. *b*. *gambiense* than with *T*. *b*. *rhodesiense* HAT samples (by 5.1% and 2.7% with IgG and IgM, respectively).

A new set of 18 antigens was included in the second round of screening. These included 14 that in the first round of screening had an IgG reactivity higher than 50% on patient sera, and 4 that were only partially soluble in the first round of screening. As the IgG reactivity to *T*. *b*. *gambiense* sera in the first round of screening was higher than IgM reactivity, and given that IgG molecules are more stable and therefore more suitable for developing a diagnostic test than IgM, subsequent rounds of screening included testing for IgG only. It was also assumed that detection of IgG would be more suitable in screening for *T*. *b*. *gambiense* HAT due to the chronic nature of this form of the disease. In addition to slot blots, antigens were also screened by ELISA in this second round in order to generate quantitative results.

**Fig 3:**
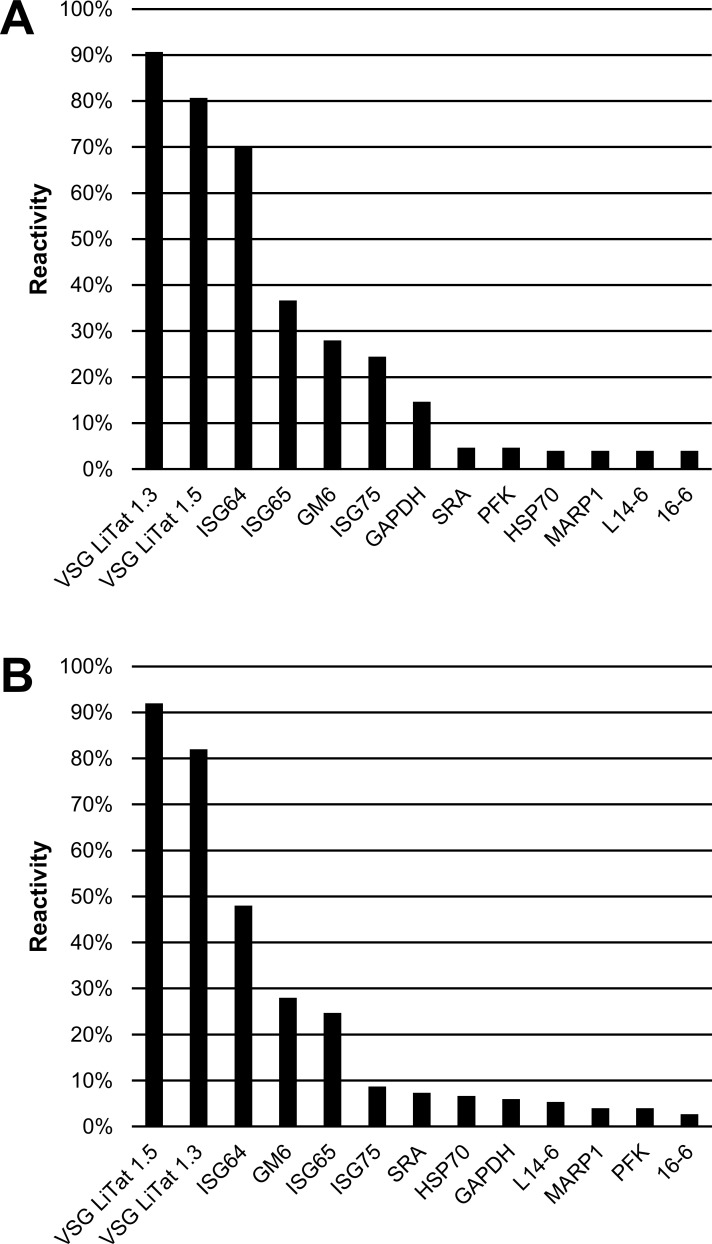
**Percentage IgG reactivity of 18 antigens on sera from *T*. *b*. *gambiense* HAT cases (N = 35) assessed by slot blot (A) and by ELISA (B) (second round of screening).** Antigens are shown in descending order of reactivity.

The reactivity of antigens to *T*. *b*. *gambiense* sera was higher using ELISA (Fig 3B) than by slot blot (Fig 3A), with an average difference of 14%. The three best antigens with both methods were VSG LiTat 1.3, VSG LiTat 1.5 and ISG64, although their ranking was different. ISG64 performed best (71%) in ELISA followed by VSG LiTat 1.3 (69%) and VSG LiTat 1.5 (69%), while VSG LiTat 1.3 was the best (60%) in slot blot, followed by VSG LiTat 1.5 (49%) and ISG64 (40%). Significant differences were observed in the ranking of the other antigens depending on the test method that was used. For example, while PFK was the fifth best antigen by slot blot, it was only the 17^th^ by ELISA.

**Fig 4:**
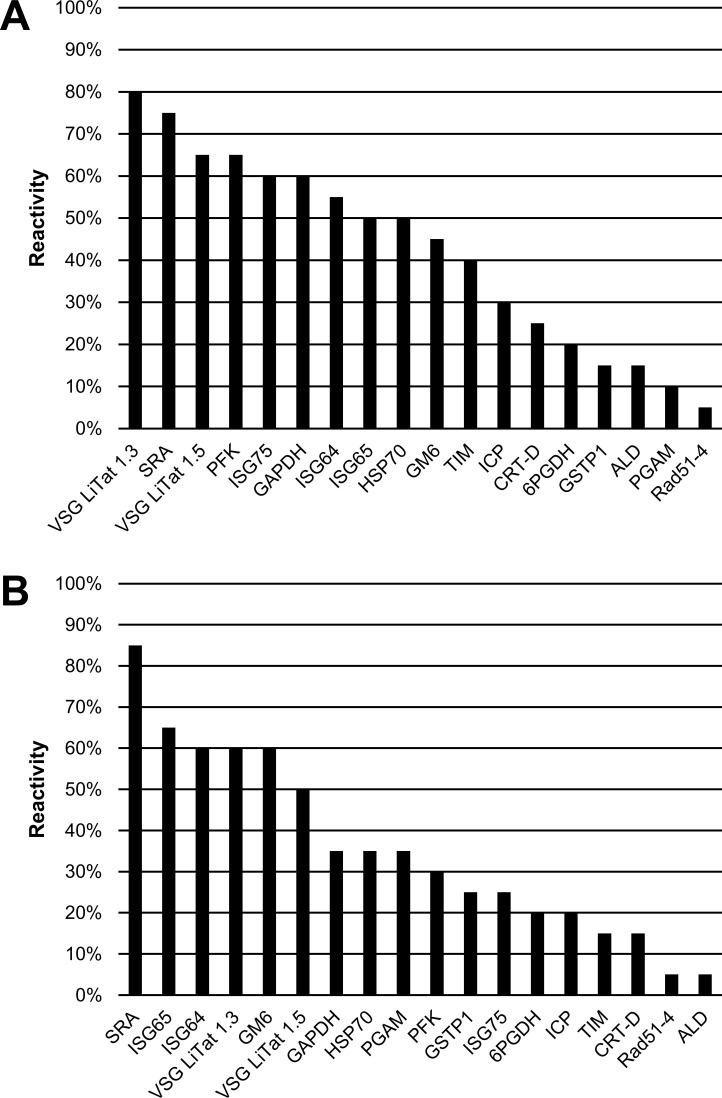
**Percentage IgG reactivity of 18 antigens on sera from *T*. *b*. *rhodesiense* HAT cases (N = 10) assessed by slot blot (A) and by ELISA (B) (second round of screening).** Antigens are shown in descending order of reactivity.

The results of screening antigens on *T*. *b*. *rhodesiense* sera are shown in Fig 4. As observed with *T*. *b*. *gambiense* sera, the antigens performed differently depending on the test method used. Using slot blotting, VSG LiTat 1.3 (80%) had the highest reactivity, followed by SRA (75%) and VSG LiTat 1.5 (65%). By ELISA, SRA performed best (85%), followed by ISG65 (65%) and ISG64 (60%). The percentage reactivity by slot blot was on average 16.6% higher with *T*. *b*. *rhodesiense* than with *T*. *b*. *gambiense* cases, which was unlike the observations obtained in the first round of screening. By contrast, reactivity by ELISA was on average 4.2% lower with *T*. *b*. *rhodesiense* than with *T*. *b*. *gambiense* sera. SRA was the antigen that exhibited the greatest difference in reactivity between the two disease forms. While the reactivity of SRA by slot blot was 75% with *T*. *b*. *rhodesiense*, it was only 17% with *T*. *b*. *gambiense*.

**Fig 5:**
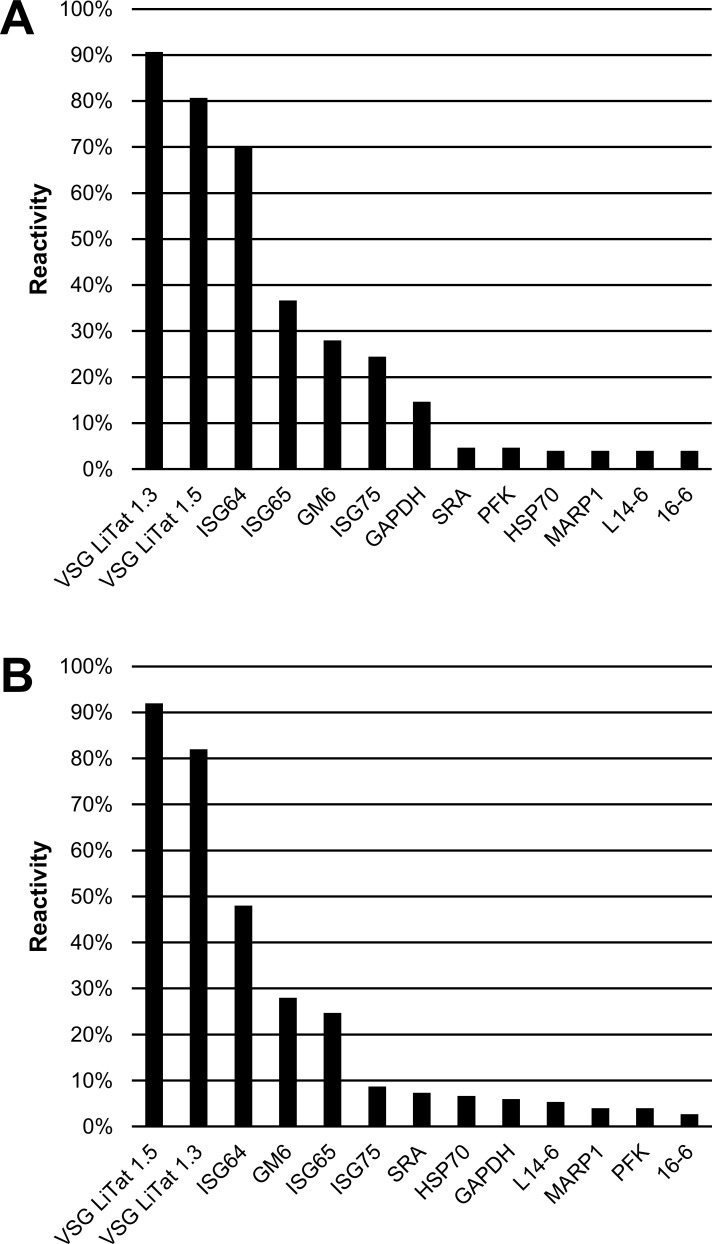
**Percentage IgG reactivity of 13 antigens on sera from *T*. *b*. *gambiense* HAT cases (N = 150) assessed by slot blot (A) and by ELISA (B) (third round of screening).** Antigens are shown in descending order of reactivity.

The third round of screening was carried out using a larger collection of clinical samples, in order to generate more significant data on the most promising antigens using both slot blot and ELISA. Thirteen antigens, including 10 that had the highest reactivity on patient sera in the second round (results of both slot blot and ELISA combined), and 3 new ones (MARP1, L14-6 and 16–6) were screened in this round. The 3 new antigens were supplied after the first two rounds of screening had been completed. Fig 5 illustrates that when the antigens were screened using *T*. *b*. *gambiense* sera, the three best antigens using both tests were the same as in the second round. Using slot blot, VSG LiTat 1.3 performed best (91%) followed by VSG LiTat 1.5 (81%) and ISG64 (70%), while by ELISA, VSG LiTat 1.5 was first (92%) followed by VSG LiTat 1.3 (82%) and ISG64 (48%). The next three antigens behaved in a similar manner with both tests, but in a different order. Using slot blot, ISG65 (37%) was followed by GM6 (28%) and ISG75 (24%), while by ELISA, GM6 (28%) was followed by ISG65 (25%) and ISG75 (9%).

**Fig 6:**
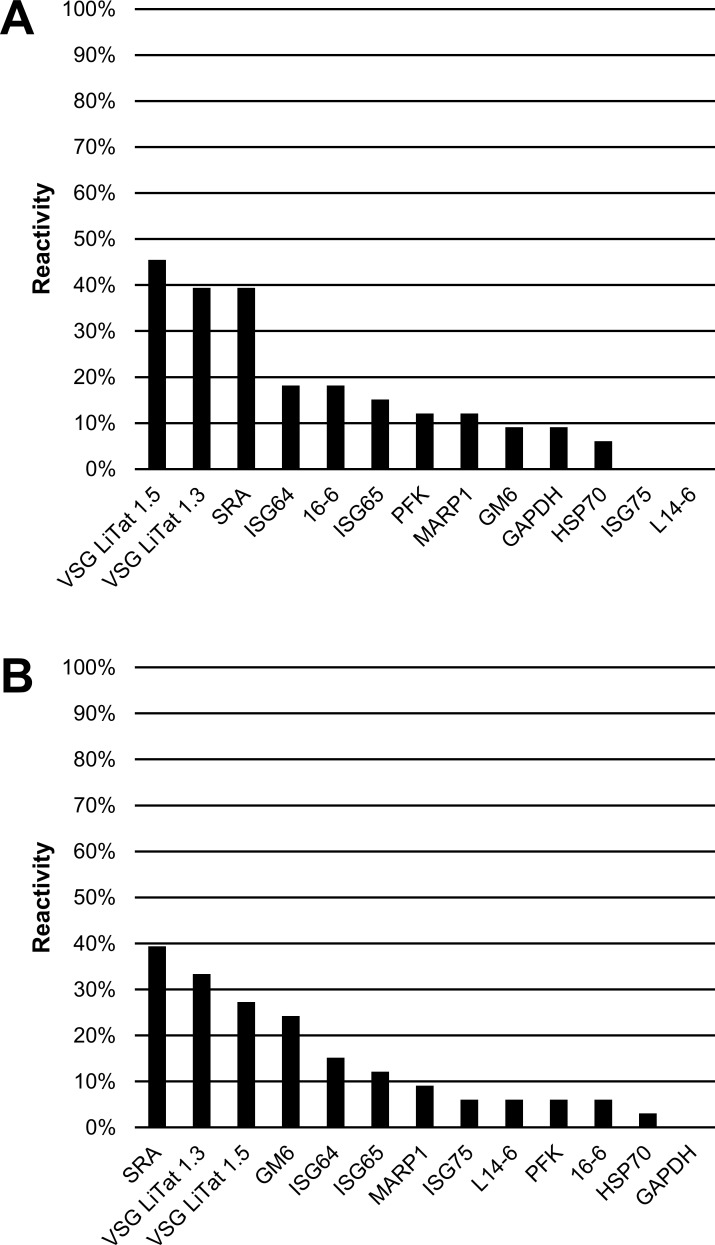
**Percentage IgG reactivity of 13 antigens on sera from *T*. *b*. *rhodesiense* HAT cases (N = 33) assessed by slot blot (A) and by ELISA (B) (third round of screening).** Antigens are shown in descending order of reactivity.

When the antigens were screened using *T*. *b*. *rhodesiense* sera, the three best antigens were the same ones irrespective of the test used (Fig 6). These were also the same antigens as observed in the second round of screening by slot blotting. According to slot blotting, VSG LiTat 1.5 (45%) was followed by VSG LiTat 1.3 (39%) and SRA (39%). By ELISA, SRA performed best (39%), followed by VSG LiTat 1.3 (33%) and VSG LiTat 1.5 (27%). In a similar manner to what was observed when the results of the first round of screening were compared, the average percentage reactivity by slot blot and by ELISA was higher with *T*. *b*. *gambiense* than with *T*. *b*. *rhodesiense* sera, with differences of 11.2% and 10.1%, respectively.

ROC analysis of the results obtained by ELISA shows that among the 13 antigens in the third round of screening, the highest accuracy for *T*. *b*. *gambiense* was achieved using VSG LiTat 1.5 (93%), followed by VSG LiTat 1.3 (79%) and ISG64 (67%) (Table 2). This analysis also indicates that if a sensitivity of 90% is considered to be acceptable, using VSG LiTat 1.5 would result in a specificity as high as 98%. Conversely, if specificity is set at 90%, a sensitivity of 97% can be reached with this antigen. VSG LiTat 1.3 could also be used to develop a test with a high specificity, but due to the lower accuracy of this antigen, there would be a stronger trade-off in sensitivity. For example, a specificity of 97% could be achieved using VSG LiTat 1.3 with a sensitivity of 70%. Using the less accurate ISG64 antigen would only result in a specificity of 91% with a sensitivity of 70%.

**Table 2:** ROC analysis of the reactivity of 13 antigens on sera from *T*. *b*. *gambiense* HAT cases (N = 150) and controls (N = 143) assessed by ELISA (third study round).

			VSG LiTat 1.5	VSG LiTat 1.3	ISG64	GM6	ISG65	ISG75	SRA	HSP70	MARP1	GAPDH	PFK	L14-6	16–6
**Maximum accuracy**		**Youden's index (%)**	93	79	67	47	47	34	25	22	10	6	NA	NA	NA
** **		**Sensitivity (%)**	96	90	85	77	81	75	68	83	91	14	NA	NA	NA
** **		**Specificity (%)**	97	89	81	70	65	59	57	39	19	92	NA	NA	NA
**Specificity (%)**	**100**	**Sensitivity (%)**	37	47	3	0	0	1	1	0	0	1	NA	NA	NA
** **	**90**	**Sensitivity (%)**	97	87	71	40	45	19	23	20	8	14	NA	NA	NA
** **	**80**	**Sensitivity (%)**	98	93	87	61	63	36	43	36	20	19	NA	NA	NA
** **	**70**	**Sensitivity (%)**	98	97	91	77	73	59	49	49	29	31	NA	NA	NA
**Sensitivity (%)**	**100**	**Specificity (%)**	3	29	47	3	11	5	0	3	3	0	NA	NA	NA
** **	**90**	**Specificity (%)**	98	89	72	31	52	41	33	28	19	18	NA	NA	NA
** **	**80**	**Specificity (%)**	98	95	85	58	67	54	39	39	29	18	NA	NA	NA
** **	**70**	**Specificity (%)**	99	97	91	75	76	65	53	51	33	41	NA	NA	NA

For each antigen, theoretical test performance was assessed by calculating Youden’s index, sensitivity and specificity using a cut-off that results in the maximum accuracy (maximum Youden’s index). The sensitivity obtained by setting a cut-off corresponding to a specificity of 100%, 90%, 80% and 70%, as well as the specificity obtained by setting a cut-off corresponding to a sensitivity of 100%, 90%, 80% and 70% are also indicated. NA: values that could not be computed by the software.

With *T*. *b*. *rhodesiense* samples, ROC analysis of the results obtained using ELISA shows that among the 13 antigens of the third round of screening, the highest accuracy was achieved with SRA (56%), followed by VSG LiTat 1.3 (43%) and VSG LiTat 1.5 (39%) (Table 3). This analysis also indicates that if a sensitivity of 90% is targeted, using SRA would only result in a specificity of 33%. Conversely, if specificity is set at 90%, a sensitivity of 61% can be reached with this antigen. Using VSG LiTat 1.3 would not be appropriate to develop a test with a high specificity, even with a strong trade-off in sensitivity. For example, a specificity of only 73% could be achieved using VSG LiTat 1.3 with a sensitivity of 70%. Using the less accurate VSG LiTat 1.5 antigen would result in a specificity of only 64% with a sensitivity of 70%.

**Table 3:** ROC analysis of the reactivity of 13 antigens on sera from *T*. *b*. *rhodesiense* HAT cases (N = 33) and controls (N = 143) assessed by ELISA (third round of screening).

			SRA	VSG LiTat 1.3	VSG LiTat 1.5	ISG65	ISG75	GM6	ISG64	16–6	MARP1	HSP70	PFK	GAPDH	L14-6
**Maximum accuracy**		**Youden's index (%)**	56	43	39	30	29	27	25	21	12	8	7	NA	NA
** **		**Sensitivity (%)**	76	70	79	76	85	48	45	30	42	18	33	NA	NA
** **		**Specificity (%)**	81	73	61	55	44	79	79	91	70	89	73	NA	NA
**Specificity (%)**	**100**	**Sensitivity (%)**	15	15	3	0	3	0	3	3	0	0	0	NA	NA
** **	**90**	**Sensitivity (%)**	61	39	36	27	12	36	27	30	12	18	12	NA	NA
** **	**80**	**Sensitivity (%)**	76	58	48	42	39	42	45	33	30	18	24	NA	NA
** **	**70**	**Sensitivity (%)**	79	70	55	55	55	48	45	45	42	33	33	NA	NA
**Sensitivity (%)**	**100**	**Specificity (%)**	19	0	8	5	5	3	6	0	1	0	0	NA	NA
** **	**90**	**Specificity (%)**	33	17	35	11	28	9	16	8	15	14	1	NA	NA
** **	**80**	**Specificity (%)**	71	55	61	45	45	27	27	21	30	14	21	NA	NA
** **	**70**	**Specificity (%)**	82	73	64	57	54	31	41	33	37	28	37	NA	NA

For each antigen, Youden’s index, sensitivity and specificity are shown using a cut-off that results in the maximum accuracy (maximum Youden’s index). The sensitivity obtained by setting a cut-off corresponding to a specificity of 100%, 90%, 80% and 70%, as well as the specificity obtained by setting a cut-off corresponding to a sensitivity of 100%, 90%, 80% and 70% are also indicated. NA: values that could not be computed by the software.

While the performance of some antigens in detecting *T*. *b*. *gambiense* HAT was very promising, combining two antigens in one test could theoretically result in a diagnostic sensitivity that is even higher. The results obtained in the third round of screening of 13 antigens were used to compute 78 possible antigen pairs that could be used to develop hypothetical tests. A combination of VSG LiTat 1.3, which detected 91% of the *T*. *b*. *gambiense* sera (Fig 3A) and ISG75 resulted in the highest reactivity (97%) using slot blot (Table 4 and S1 Table). This was followed by VSG LiTat 1.3 combined with ISG64 (95%) and by VSG LiTat 1.3 combined with either GM6 or VSG LiTat 1.5 (93%). Using ELISA (S2 Table), the best antigen pair to detect *T*. *b*. *gambiense* sera was VSG LiTat 1.5 and VSG LiTat 1.3 (95%), followed by VSG LiTat 1.5 and 16–6 (94%), and by VSG LiTat 1.5 combined with either GM6 or ISG64 (93%). The reactivity of these pairs in ELISA was higher than using VSG LiTat 1.5 alone (92%, Fig 3B).

**Table 4:** Percentage IgG reactivity of the five most reactive antigen pairs as well as the five most reactive individual antigens on sera from *T*. *b*. *gambiense* HAT cases (N = 150) and *T*.*b*. *rhodesiense* HAT cases (N = 33) assessed by slot blot (third round of screening).

Antigen pair/ antigen	*T*. *b*. *gambiense* reactivity (%)	Antigen pair/ antigen	*T*. *b*. *rhodesiense* reactivity (%)
VSG LiTat 1.3 & ISG75	97	VSG LiTat 1.3 & SRA	67
VSG LiTat 1.3 & ISG64	95	VSG LiTat 1.5 & SRA	67
VSG LiTat 1.3 & GM6	93	VSG LiTat 1.5 & ISG64	55
VSG LiTat 1.5 & VSG LiTat 1.3	93	MARP1 & VSG LiTat 1.5	55
MARP1 & VSG LiTat 1.3	92	VSG LiTat 1.3 & ISG64	52
VSG LiTat 1.3	91	VSG LiTat 1.5	45
VSG LiTat 1.5	81	VSG LiTat 1.3	39
ISG64	70	SRA	39
ISG65	37	ISG64	18
GM6	28	16–6	18

Antigen pairs and individual antigens are shown in descending order of reactivity.

A combination of antigens also resulted in a stronger reactivity on *T*. *b*. *rhodesiense* sera. Reactivity increased to 67% when SRA was combined with either VSG LiTat 1.3 or VSG LiTat 1.5 in slot blot (Table 4 and S3 Table), and to 61% when SRA was combined with either GM6 or VSG LiTat 1.3 in ELISA (S4 Table). These were significant improvements when compared to the reactivity of the best single antigens, which were VSG LiTat 1.5 (45%) and SRA (39%) in slot blot and ELISA respectively.

## Discussion

This study comprised the first systematic, independent assessment of the diagnostic potential of a large panel of antigens that could potentially be used for development of a rapid diagnostic test (RDT) for HAT. Since this work was completed, a number of RDTs have actually been developed, and are briefly discussed below. A number of very promising antigens were identified, both for *gambiense* and for *rhodesiense* HAT. While there were many similarities in the results of the three rounds of screening, there were also a number of important differences, both in the ranking of antigens and in their absolute reactivity. The reactivity of the best antigens was significantly higher in the first and third rounds than in the second round, and differences between antigens were also more pronounced in the third round. These differences between study rounds might be explained by two factors. First, the sample size was significantly larger in the third round compared to other rounds, which enabled more accurate measurements. Second, the clinical samples used in the first and second rounds had been stored for lengthy periods, during which they could have gone through multiple unknown cycles of freezing and thawing, which could have caused some loss of immunoreactivity. Significant degradation was in fact observed in some of the samples that were supplied for the second round. This may have resulted in a biased assessment of the reactivity of some antigens and possibly exclusion of some of them from subsequent rounds. By contrast, samples used in the third round were collected recently, and subjected to a maximum of two rounds of freezing and thawing. Therefore, results from the third round of testing should be considered as a more reliable indicator of the reactivity of antigens. Also, results obtained with *gambiense* samples are likely to be more reliable than those obtained with *rhodesiense*, as they were based on a much larger sample set.

The antigens that were most reactive were proteins that are known to be localized extracellularly, on the surface of parasites, which may explain why strong immunoreactivity was observed in plasma samples from infected patients. In particular, VSG LiTat 1.3 and VSG LiTat 1.5, which belong to the group of VSGs that cover the external surface of the bloodstream form of *T*. *brucei* [39], were found to be the two most reactive antigens against *gambiense* samples, and among the three most reactive antigens against *rhodesiense* samples. Noteworthy, *T*. *b*. *gambiense* clones expressing these VSGs were also among the most sensitive for detecting *gambiense* patients using immune trypanolysis [40]. However, VSGs have been reported to exhibit some heterogeneity in their expression among parasite isolates, and could therefore present a risk in terms of sensitivity if they are included in a diagnostic test. In particular, the gene encoding VSG LiTat 1.3 was found to be absent in some *T*. *b*. *gambiense* isolates from Cameroon [41]. Other surface proteins such as ISG64, ISG65 and to a lesser extent ISG75 were also quite reactive, especially to samples from *gambiense* patients. In contrast, proteins that are known to be intracellular, and therefore less accessible to the host’s immune system, exhibited a very low reactivity. These include enzymes of the glycolytic pathway (GAPDH and PFK), a cytoskeleton-associated protein (MARP1), a pyrimidine biosynthetic enzyme (DHODH) and a chaperone (HSP70). However, there were also exceptions to this perhaps over-simplistic distinction. In particular, SRA exhibited a strong reactivity to *rhodesiense* samples, although it has been shown to be an intracellular protein localized to the lysosome and the endocytic pathway [42]. It is also possible that the observed reactivity of SRA would relate to cross-reacting antibodies that were actually generated against VSGs, since SRA is closely related to VSGs [43]. In addition, GM6, which is located on flagellum attachment zone fibres connecting microtubules to the flagellum [18], was moderately reactive to *gambiense* samples. While they may not be very useful for serodiagnosis, some of the intracellular proteins that were tested in this study might have utility as a means to detect response to therapy when parasites are lysed after cidal effects of drug treatment. The fact that the human protein GSTP1 exhibited significant reactivity to both *gambiense* and *rhodesiense* samples might be explained by non-specific activation of B lymphocytes, which has been reported to occur during trypanosomal infections in mice and in cattle [44–47]. This phenomenon might also be responsible for the significant reactivity of SRA to *gambiense* samples that was found in this study.

The chronic nature of *gambiense* HAT could provide an explanation for the observation that IgG reactivity was on average higher with *gambiense* than with *rhodesiense* samples, as patients infected with *T*. *b*. *gambiense* are likely to have been diagnosed and sampled after a significantly longer infection time than in the case of the more acute *T*. *b*. *rhodesiense* infections. On the other hand, *rhodesiense* patients may not have had sufficient time to mount a strong IgG response comparable to that observed in *gambiense* patients. The fact that the difference in reactivity between the two forms of the disease was less pronounced in the case of IgM is consistent with this explanation, as IgM production occurs earlier in infections, and large amounts of this class of antibody may be produced prior to diagnosis of *gambiense* or *rhodesiense* patients. An alternative explanation for this less pronounced difference could be the generally lower specificity of IgM in comparison to IgG antibodies.

The reactivity values obtained in the three rounds using a fixed cut-off (mean reactivity of controls plus 2 standard deviations) correspond to sensitivity values that would theoretically be obtained with a test that is calibrated as having a specificity of 95.4%, assuming a normal distribution [48]. The ROC analysis that was performed in the third round allowed better appreciation of the full diagnostic potential of antigens by calculating the accuracy and the relationship between sensitivity and specificity of each antigen. Although the ranking of antigens according to accuracy (Youden’s index) was very similar to that obtained with a fixed cut-off by ELISA, this ROC analysis provides additional information and shows that using the same antigen, tests with very different diagnostic properties could be developed. For example, using VSG LiTat 1.5 on *gambiense* samples, a test that is highly specific (98%) but moderately sensitive (90%) could be developed, as well as a test that is highly sensitive (97%) but moderately specific (90%). Therefore, information on the intended use of a test, including the target population, disease incidence, setting, diagnostic algorithm as well as available treatments, will need to be considered carefully when defining target specifications to be achieved in test development using these antigens.

As no single antigen assessed in this study reacted with all the positive sera, combining two or more antigens might be necessary in order to develop a test with a sensitivity that would be sufficiently high for effective disease control and elimination. An example is a combination of VSG LiTat 1.3 and VSG LiTat 1.5, which performed best by slot blot on *T*. *b*. *gambiense* sera. Since slot blot uses a nitrocellulose matrix that is similar to that used in RDTs, these antigens were considered as the best candidates for developing an RDT. Indeed, since this study was carried out, two RDTs for *gambiense* HAT have been developed and commercialized, both using these two antigens. One is the HAT Sero-*K*-SeT manufactured by Coris BioConcept (Belgium), with a reported sensitivity of 93.9% and a specificity of 99.0% using archived sera [23], as well as a sensitivity of 98.5% and a specificity of 98.6% in a prospective passive screening trial [49]. The other is the SD BIOLINE HAT produced by Alere/Standard Diagnostics (South Korea). A study that compared the performance of these two RDTs on stored plasma originating from Guinea and Côte d’Ivoire reported sensitivities of 99.1% and 99.6%, and specificities of 88.3% and 87.9% for the HAT Sero-K-SeT and the SD BIOLINE HAT, respectively [50]. A prototype version of the SD BIOLINE HAT was also evaluated in a prospective study in Angola, the DRC and the Central African Republic [51], without pre-selecting participants using CATT. This study concluded that the sensitivity of the RDT was not statistically different from the sensitivity of CATT when performed on whole blood, while its specificity was 1.3% lower than that of CATT. Taken together, these results demonstrate that the methodology that we used in the present study was appropriate in identifying antigens with a high potential for development of an RDT for HAT.

Most of the antigens were found to react to both *gambiense* and *rhodesiense* infections, and the antigens that exhibited a strong reactivity with samples from one form of the disease were also reactive to the other form. These results are consistent with the extremely high level of genomic similarity that has been reported between *T*. *b*. *gambiense* and *T*. *b*. *rhodesiense* [52,53]. SRA was an exception, since this antigen was much more reactive to *rhodesiense* than to *gambiense* samples. This finding is in agreement with the fact that SRA is a protein that is only expressed by *T*. *b*. *rhodesiense* and not by *T*. *b*. *gambiense* [21,22]. Although developing an RDT that would detect both forms of disease using some of the antigens included in this panel could be considered, it seems that even if two antigens are combined, the resulting sensitivity for *rhodesiense* would not be sufficiently high. Further research is therefore necessary to identify other antigens for use in developing a test for *rhodesiense* HAT. One approach might be to evaluate the diagnostic potential of the 254 proteins that were identified by Eyford *et al*. [54] in the plasma from patients infected with *T*. *b*. *rhodesiense*. Although *gambiense* and *rhodesiense* HAT are found in discrete geographical foci [55], there have been concerns that the two disease forms may eventually coexist in the same region. In particular, outbreaks of *rhodesiense* HAT in previously unaffected districts of Uganda resulted in a significant expansion of the *rhodesiense* HAT endemic region from the southeast towards the northwestern region of the country, posing a risk of overlap with the other disease form [56]. In such an event, an RDT that would be specific for *T*. *b*. *rhodesiense* infections would be very useful, since treatments differ between the two disease forms [57]. It would also be worth testing samples from *rhodesiense* patients with the RDTs that have been developed for *gambiense* HAT, as these tests might also prove useful as screening tools for this form of disease. Moreover, it would be interesting to assess the diagnostic potential of the *T*. *b*. *gambiense*-specific TgsGP protein [58,59], which was not available for inclusion in this study.

The results from the second and third rounds of screening were obtained using two different and complementary methods. Assessment by slot blotting gives an indication of the performance of antigens on a nitrocellulose membrane, which is a commonly used solid phase platform in lateral-flow RDTs. On the other hand, ELISA relies on photometric measurements that should allow for a more precise quantification of the reactivity of antigens. Although there were some differences in the ranking of antigens using these two methods, the overall pattern of results for both slot blotting and ELISA was remarkably similar, reinforcing the validity of the results generated in this study. However, some differences in reactivity were found using these two methods. For instance, ISG75 and GAPDH exhibited a much higher reactivity in slot blot than in ELISA. Although the reasons for such differences were not investigated, binding properties of different antigens to the two matrices are likely to play a role, which may result in distinct affinity as well as different orientation or denaturation of antigens. When interpreting these results, it is therefore critical to also take into account the matrix on which the antigens would eventually be applied in a serological test. In our case, the target was to develop an RDT with a nitrocellulose matrix, and therefore the slot blot results were given higher consideration.

While efforts were made to be as comprehensive as possible and include all the antigens that had been previously reported as having a diagnostic potential, this work only included 35 antigens. It is possible that other antigens that were either not included or had not been identified at that time could perform equally well or better than assessed in the present study. For example, the flagellar calcium-binding protein TB-17 [20,60], the Gene Related to Expression Site Associated Gene (GRESAG) 4, and transferrin receptor subunits ESAG 6 and 7 have also been suggested as potential candidates [20]. Since the most reactive antigens include the native VSG LiTat 1.3 and VSG LiTat 1.5 antigens, which are costly and difficult to produce, one approach might be to generate recombinant versions of these proteins. This was attempted by Rogé *et al*. [61], who expressed the immunogenic N-terminal part of these antigens in *Pichia pastoris*, and by Rooney *et al*. [62], who expressed these antigens and ISG65 in *Leishmania tarentolae*. While evaluation of these recombinant antigens by ELISA gave very promising results, their diagnostic potential in an RDT format remains to be confirmed. Another approach has been to produce synthetic peptide mimotopes corresponding to epitopes of VSG LiTat 1.3 and VSG LiTat 1.5; however, the diagnostic accuracy of these peptides was found to be lower than that of full-length native antigens [63–65].

An important limitation of this study was in the selection of the clinical samples that were used to screen the antigens. All *gambiense* HAT cases were identified following screening using the CATT test. CATT reagents consist of lyophilized bloodstream form *T*. *b*. *gambiense* parasites expressing VSG LiTat 1.3 [4]. This means that by definition, all samples from *gambiense* HAT cases were sero-reactive to VSG LiTat 1.3. This selection bias could explain why this antigen performed so well in this study. In addition, it is conceivable that samples from other *gambiense* HAT cases with a different serological profile and that would potentially be more reactive to other antigens could have been missed during screening with CATT. Similarly, all controls originating from *gambiense* endemic countries had to be negative with CATT. This is likely to have introduced a bias resulting in an over-estimate of the specificity of antigens that are related to CATT, such as VSG LiTat 1.3. It is therefore possible that the results of this study could have been significantly different if samples had not been pre-selected using any serological test. Unfortunately, we could not get access to samples that were not pre-selected by CATT, since virtually all the prospective studies involving sample collection that have been conducted over the last few decades involved selection of participants on the basis of CATT results. Future attempts to evaluate antigens for their potential in diagnosis of HAT would benefit from new prospective collections of clinical samples whose selection would not rely on serological tests, but which could take advantage of new diagnostic tools, such as the molecular detection tests that have recently been made available [66].

## Supporting Information

S1 Table Percentage IgG reactivity of antigen pairs on sera from *T*. *b*. *gambiense* HAT cases assessed by slot blot. The 78 possible antigen pairs computed with the 13 antigens from the third round of screening are shown in descending order of reactivity.
(PDF)

S2 Table Percentage IgG reactivity of antigen pairs on sera from *T*. *b*. *gambiense* HAT cases assessed by ELISA. The 78 possible antigen pairs computed with the 13 antigens from the third round of screening are shown in descending order of reactivity.
(PDF)

S3 Table Percentage IgG reactivity of antigen pairs on sera from *T*. *b*. *rhodesiense* HAT cases assessed by slot blot. The 78 possible antigen pairs computed with the 13 antigens from the third round of screening are shown in descending order of reactivity.
(PDF)

S4 Table Percentage IgG reactivity of antigen pairs on sera from *T*. *b*. *rhodesiense* HAT cases assessed by ELISA. The 78 possible antigen pairs computed with the 13 antigens from the third round of screening are shown in descending order of reactivity.
(PDF)
