# Supplementary material for: Evaluation of Antigens for Development of a Serological Test for Human African Trypanosomiasis
Source: PLoS One. 2016 Dec 9;11(12):e0168074. doi: 10.1371/journal.pone.0168074 (PMC5148118; doi:10.1371/journal.pone.0168074)
Supplement: S1 Table — The 78 possible antigen pairs computed with the 13 antigens from the third round of screening are shown in descending order of reactivity. (PDF) [file pone.0168074.s001.pdf]

Antigen pair	Reactivity (%)
VSG LiTat 1.3 & ISG75	97
VSG LiTat 1.3 & ISG64	95
VSG LiTat 1.3 & GM6	93
VSG LiTat 1.5 & VSG LiTat 1.3	93
MARP1 & VSG LiTat 1.3	92
16-6 & VSG LiTat 1.3	92
VSG LiTat 1.3 & ISG65	91
HSP70 & VSG LiTat 1.3	91
PFK & VSG LiTat 1.3	91
GAPDH & VSG LiTat 1.3	91
VSG LiTat 1.3 & SRA	91
L14-6 & VSG LiTat 1.3	91
VSG LiTat 1.5 & ISG64	88
VSG LiTat 1.5 & ISG75	87
VSG LiTat 1.5 & GM6	85
VSG LiTat 1.5 & ISG65	83
GAPDH & VSG LiTat 1.5	82
MARP1 & VSG LiTat 1.5	82
16-6 & VSG LiTat 1.5	82
VSG LiTat 1.5 & SRA	81
HSP70 & VSG LiTat 1.5	81
PFK & VSG LiTat 1.5	81
L14-6 & VSG LiTat 1.5	81
ISG75 & ISG64	77
ISG64 & GM6	74
GAPDH & ISG64	73
ISG65 & ISG64	72
L14-6 & ISG64	72
16-6 & ISG64	71
HSP70 & ISG64	71
MARP1 & ISG64	71
SRA & ISG64	70
PFK & ISG64	70
ISG75 & ISG65	54
ISG65 & GM6	49
GAPDH & ISG65	45
ISG75 & GM6	44
HSP70 & ISG65	39
PFK & ISG65	39
L14-6 & ISG65	39
SRA & ISG65	38
MARP1 & ISG65	38
GAPDH & ISG75	38
16-6 & ISG65	37
GAPDH & GM6	36
PFK & GM6	31
L14-6 & GM6	31
MARP1 & GM6	31
SRA & GM6	30
HSP70 & GM6	30
16-6 & GM6	30
SRA & ISG75	30
PFK & ISG75	30
L14-6 & ISG75	29
HSP70 & ISG75	28
16-6 & ISG75	28
MARP1 & ISG75	27
GAPDH & PFK	18
L14-6 & GAPDH	18
GAPDH & SRA	17
MARP1 & GAPDH	17
GAPDH & HSP70	17
16-6 & GAPDH	17
HSP70 & SRA	9
PFK & SRA	9
MARP1 & SRA	9
L14-6 & SRA	9
16-6 & SRA	9
L14-6 & PFK	9
16-6 & PFK	9
PFK & HSP70	8
L14-6 & HSP70	8
L14-6 & MARP1	8
16-6 & L14-6	8
MARP1 & HSP70	7
16-6 & HSP70	7
16-6 & MARP1	7
MARP1 & PFK	7
