# Supplementary material for: Evaluation of Antigens for Development of a Serological Test for Human African Trypanosomiasis
Source: PLoS One. 2016 Dec 9;11(12):e0168074. doi: 10.1371/journal.pone.0168074 (PMC5148118; doi:10.1371/journal.pone.0168074)
Supplement: S2 Table — The 78 possible antigen pairs computed with the 13 antigens from the third round of screening are shown in descending order of reactivity. (PDF) [file pone.0168074.s002.pdf]

Antigen pair	Reactivity (%)	Antigen pair	Reactivity (%)	Antigen pair	Reactivity (%)
VSG LiTat 1.5 & VSG LiTat 1.3	95	PFK & ISG64	50	HSP70 & ISG75	13
16-6 & VSG LiTat 1.5	94	L14-6 & ISG64	50	GAPDH & ISG75	13
VSG LiTat 1.5 & GM6	93	16-6 & ISG64	50	PFK & ISG75	12
VSG LiTat 1.5 & ISG64	93	SRA & ISG64	49	HSP70 & SRA	12
L14-6 & VSG LiTat 1.5	93	HSP70 & ISG64	49	L14-6 & SRA	12
VSG LiTat 1.5 & ISG65	93	MARP1 & ISG64	49	MARP1 & ISG75	11
MARP1 & VSG LiTat 1.5	93	GAPDH & ISG64	49	16-6 & ISG75	11
VSG LiTat 1.5 & ISG75	92	ISG65 & GM6	42	GAPDH & HSP70	11
VSG LiTat 1.5 & SRA	92	ISG75 & GM6	33	L14-6 & HSP70	11
HSP70 & VSG LiTat 1.5	92	SRA & GM6	33	PFK & SRA	11
PFK & VSG LiTat 1.5	92	L14-6 & GM6	33	GAPDH & SRA	11
GAPDH & VSG LiTat 1.5	92	GAPDH & GM6	32	MARP1 & SRA	11
VSG LiTat 1.3 & GM6	85	HSP70 & GM6	31	PFK & HSP70	11
16-6 & VSG LiTat 1.3	84	PFK & GM6	31	L14-6 & GAPDH	11
VSG LiTat 1.3 & ISG64	83	MARP1 & GM6	31	16-6 & SRA	10
VSG LiTat 1.3 & ISG65	83	16-6 & GM6	30	GAPDH & PFK	10
HSP70 & VSG LiTat 1.3	83	SRA & ISG65	30	MARP1 & HSP70	9
PFK & VSG LiTat 1.3	83	GAPDH & ISG65	29	L14-6 & PFK	9
MARP1 & VSG LiTat 1.3	83	L14-6 & ISG65	29	MARP1 & GAPDH	9
L14-6 & VSG LiTat 1.3	83	ISG75 & ISG65	28	16-6 & HSP70	9
VSG LiTat 1.3 & ISG75	82	HSP70 & ISG65	28	16-6 & GAPDH	9
VSG LiTat 1.3 & SRA	82	MARP1 & ISG65	28	MARP1 & PFK	8
GAPDH & VSG LiTat 1.3	82	PFK & ISG65	27	L14-6 & MARP1	7
ISG64 & GM6	57	16-6 & ISG65	27	16-6 & L14-6	7
ISG65 & ISG64	53	SRA & ISG75	15	16-6 & PFK	7
ISG75 & ISG64	50	L14-6 & ISG75	14	16-6 & MARP1	6
