# Supplementary material for: Evaluation of Antigens for Development of a Serological Test for Human African Trypanosomiasis
Source: PLoS One. 2016 Dec 9;11(12):e0168074. doi: 10.1371/journal.pone.0168074 (PMC5148118; doi:10.1371/journal.pone.0168074)
Supplement: S3 Table — The 78 possible antigen pairs computed with the 13 antigens from the third round of screening are shown in descending order of reactivity. (PDF) [file pone.0168074.s003.pdf]

Antigen pair	Reactivity (%)
VSG LiTat 1.3 & SRA	67
VSG LiTat 1.5 & SRA	67
VSG LiTat 1.5 & ISG64	55
MARP1 & VSG LiTat 1.5	55
VSG LiTat 1.3 & ISG64	52
16-6 & SRA	52
MARP1 & VSG LiTat 1.3	52
16-6 & VSG LiTat 1.5	52
VSG LiTat 1.3 & ISG65	48
VSG LiTat 1.5 & ISG65	48
PFK & SRA	48
16-6 & VSG LiTat 1.3	48
GAPDH & VSG LiTat 1.5	48
VSG LiTat 1.5 & GM6	45
SRA & ISG64	45
VSG LiTat 1.5 & ISG75	45
GAPDH & SRA	45
MARP1 & SRA	45
VSG LiTat 1.5 & VSG LiTat 1.3	45
HSP70 & VSG LiTat 1.5	45
PFK & VSG LiTat 1.5	45
L14-6 & VSG LiTat 1.5	45
SRA & GM6	42
HSP70 & VSG LiTat 1.3	42
PFK & VSG LiTat 1.3	42
GAPDH & VSG LiTat 1.3	42

Antigen pair	Reactivity (%)
VSG LiTat 1.3 & GM6	39
SRA & ISG65	39
SRA & ISG75	39
VSG LiTat 1.3 & ISG75	39
HSP70 & SRA	39
L14-6 & SRA	39
L14-6 & VSG LiTat 1.3	39
16-6 & ISG64	36
PFK & ISG64	30
MARP1 & ISG64	30
16-6 & ISG65	30
ISG64 & GM6	27
GAPDH & ISG64	27
ISG65 & GM6	24
16-6 & GM6	24
ISG65 & ISG64	24
HSP70 & ISG64	24
PFK & ISG65	24
GAPDH & ISG65	24
MARP1 & ISG65	24
16-6 & GAPDH	24
16-6 & MARP1	24
PFK & GM6	21
MARP1 & GM6	21
GAPDH & PFK	21
MARP1 & PFK	21

Antigen pair	Reactivity (%)
16-6 & PFK	21
MARP1 & GAPDH	21
ISG75 & ISG64	18
L14-6 & ISG64	18
HSP70 & ISG65	18
16-6 & ISG75	18
16-6 & HSP70	18
16-6 & L14-6	18
GAPDH & GM6	15
ISG75 & ISG65	15
L14-6 & ISG65	15
PFK & HSP70	15
MARP1 & HSP70	15
HSP70 & GM6	12
PFK & ISG75	12
MARP1 & ISG75	12
GAPDH & HSP70	12
L14-6 & PFK	12
L14-6 & MARP1	12
ISG75 & GM6	9
L14-6 & GM6	9
GAPDH & ISG75	9
L14-6 & GAPDH	9
HSP70 & ISG75	6
L14-6 & HSP70	6
L14-6 & ISG75	0
