# Supplementary material for: Evaluation of Antigens for Development of a Serological Test for Human African Trypanosomiasis
Source: PLoS One. 2016 Dec 9;11(12):e0168074. doi: 10.1371/journal.pone.0168074 (PMC5148118; doi:10.1371/journal.pone.0168074)
Supplement: S4 Table — The 78 possible antigen pairs computed with the 13 antigens from the third round of screening are shown in descending order of reactivity. (PDF) [file pone.0168074.s004.pdf]

Antigen pair	Reactivity (%)
SRA & GM6	61
VSG LiTat 1.3 & SRA	61
VSG LiTat 1.5 & SRA	52
SRA & ISG75	45
PFK & SRA	45
MARP1 & SRA	45
L14-6 & SRA	45
16-6 & SRA	45
VSG LiTat 1.3 & GM6	42
SRA & ISG64	42
VSG LiTat 1.3 & ISG65	42
HSP70 & SRA	42
VSG LiTat 1.3 & ISG64	39
SRA & ISG65	39
VSG LiTat 1.3 & ISG75	39
GAPDH & SRA	39
VSG LiTat 1.5 & VSG LiTat 1.3	39
MARP1 & VSG LiTat 1.3	39
ISG65 & GM6	36
VSG LiTat 1.5 & GM6	36
HSP70 & VSG LiTat 1.3	36
PFK & VSG LiTat 1.3	36
L14-6 & VSG LiTat 1.3	36
16-6 & VSG LiTat 1.3	36
MARP1 & VSG LiTat 1.5	36
ISG64 & GM6	33

Antigen pair	Reactivity (%)
VSG LiTat 1.5 & ISG75	33
GAPDH & VSG LiTat 1.3	33
MARP1 & GM6	30
VSG LiTat 1.5 & ISG64	30
VSG LiTat 1.5 & ISG65	30
HSP70 & VSG LiTat 1.5	30
PFK & VSG LiTat 1.5	30
L14-6 & VSG LiTat 1.5	30
16-6 & VSG LiTat 1.5	30
HSP70 & GM6	27
L14-6 & GM6	27
16-6 & GM6	27
GAPDH & VSG LiTat 1.5	27
ISG75 & GM6	24
PFK & GM6	24
GAPDH & GM6	24
MARP1 & ISG64	24
ISG65 & ISG64	21
ISG75 & ISG64	21
PFK & ISG64	21
L14-6 & ISG64	21
MARP1 & ISG65	21
HSP70 & ISG64	18
16-6 & ISG64	18
ISG75 & ISG65	18
PFK & ISG65	18

Antigen pair	Reactivity (%)
L14-6 & ISG65	18
16-6 & ISG65	18
GAPDH & ISG64	15
HSP70 & ISG65	15
MARP1 & PFK	15
L14-6 & MARP1	15
16-6 & MARP1	15
GAPDH & ISG65	12
PFK & ISG75	12
MARP1 & ISG75	12
L14-6 & ISG75	12
16-6 & ISG75	12
MARP1 & HSP70	12
16-6 & PFK	12
16-6 & L14-6	12
HSP70 & ISG75	9
PFK & HSP70	9
L14-6 & HSP70	9
16-6 & HSP70	9
L14-6 & PFK	9
MARP1 & GAPDH	9
GAPDH & ISG75	6
GAPDH & PFK	6
L14-6 & GAPDH	6
16-6 & GAPDH	6
GAPDH & HSP70	3
